# The genetic landscape of the T cell non-inflamed tumor microenvironment in human solid tumors

**DOI:** 10.1186/2051-1426-3-S2-P97

**Published:** 2015-11-04

**Authors:** Jason J Luke, Stefani Spranger, Riyue Bao, Jorge Andrade, Thomas F Gajewski

**Affiliations:** 1 University of Chicago, Chicago, IL, USA; 2 University of Chicago Medicine, Chicago, IL, USA

## Background

A subset of patients with cancer have evidence of a spontaneous anti-tumor immune response and T cell infiltration into tumor sites. This has prognostic significance and is associated with clinical response to immunotherapeutics in multiple cancers. It has been observed that high mutational load in a tumor also correlates with response to modern immunotherapies though definitive mechanistic data has yet to be shown. Molecular mechanisms underpinning the absence of a T cell response are beginning to be understood with identification specifically of the WNT/β-catenin pathway playing a major role in melanoma.

## Methods

To better understand the genetic landscape of T cell-inflamed human solid tumors and to investigate molecular pathways that may mediate immune-exclusion, we performed an *in silico* analysis of The Cancer Genome Atlas (TCGA), sorting gene expression profiling and DNA sequencing data across all tumor types included. For each tumor, RNAseq data was subjected to unsupervised clustering on ~15,000 genes expressed in at least 50% of the samples.

## Results

We discovered 160 genes concordantly clustered with a previously identified 13 gene immune signature across all cancers. Subsequent consensus sample clustering using the concordant genes facilitated division of each tumor into either a high, medium or low T cell-inflamed status. Simultaneously, non-synonymous exonic somatic mutations were retrieved from DNA sequencing data allowing mutational load to be associated with the presence or absence of a T cell-inflamed phenotype in individual samples. Pathway analysis was performed to identify molecular signaling pathways showing activation in low versus high T cell-inflamed tumors.

Overall the analysis identified the T cell-inflamed phenotype as being present in approximately 33% of all samples within TCGA with clear cell kidney cancer demonstrating the highest median score and uveal melanoma demonstrating the lowest score. While there was some trend between tumors known to have high mutational load and higher incidence of the T cell-inflamed tumor microenvironment, this correlation was imperfect, and within individual tumors there was no difference in mutational load between high T cell-inflamed tumors and low T cell-inflamed tumors. Pathways analysis again confirmed WNT/β-catenin pathway activation in non-T cell-inflamed melanomas as well as other non-T cell-inflamed cancers. Pathways of interest by individual tumor type are under further investigation.

## Conclusions

These data suggest that spontaneous anti-tumor immune priming is not driven by mutational load alone. Additionally, it appears likely that tumor-intrinsic molecular pathways will be identified in many tumor types which may suggest rational combination therapy approaches for the future.

